# Soft-Sensing Regression Model: From Sensor to Wafer Metrology Forecasting

**DOI:** 10.3390/s23208363

**Published:** 2023-10-10

**Authors:** Angzhi Fan, Yu Huang, Fei Xu, Sthitie Bom

**Affiliations:** 1 Department of Statistics, University of Chicago, Chicago, IL 60637, USA; 2 Seagate Technology, Fremont, CA 94538, USA; yu.1.huang@seagate.com; 3 Department of Astronomy and Astrophysics, University of Chicago, Chicago, IL 60637, USA; feixu@uchicago.edu; 4 Seagate Technology, Bloomington, MN 55435, USA; sthitie.e.bom@seagate.com

**Keywords:** data processing, artificial intelligence, wafer manufacturing

## Abstract

The semiconductor industry is one of the most technology-evolving and capital-intensive market sectors. Effective inspection and metrology are necessary to improve product yield, increase product quality and reduce costs. In recent years, many types of semiconductor manufacturing equipments have been equipped with sensors to facilitate real-time monitoring of the production processes. These production-state and equipment-state sensor data provide an opportunity to practice machine-learning technologies in various domains, such as anomaly/fault detection, maintenance scheduling, quality prediction, etc. In this work, we focus on the soft-sensing regression problem in metrology systems, which uses sensor data collected during wafer processing steps to predict impending inspection measurements that used to be measured in wafer inspection and metrology systems. We proposed a regressor based on Long Short-term Memory network and devised two distinct loss functions for the purpose of the training model. Although the assessment of our prediction errors by engineers is subjective, a novel piece-wise evaluation metric was introduced to evaluate model accuracy in a mathematical way. Our experimental results showcased that the proposed model is capable of achieving both accurate and early prediction across various types of inspections in complicated manufacturing processes.

## 1. Introduction

The strategic importance of AI technologies in the semiconductor industry lies in their capacity to minimize capital requirements and enhance cycle time and yields. This presents a considerable opportunity to introduce pioneering machine learning methods that can optimize our manufacturing processes and improve their efficiency significantly. Among these promising techniques is virtual metrology (VM), also known as soft-sensing models or virtual sensors, which plays a vital role in effectively monitoring industrial processes. In broad essence, soft-sensing models refer to inferential models that employ *easy-to-measure* variables (e.g., online available sensors) to dynamically estimate *hard-to-measure* variables (e.g., quality variables) in real-time. Nonetheless, the progress in creating data-driven soft-sensing models, particularly those based on machine learning, has been notably sluggish and erratic. The primary issue lies in the lack of scalability in the majority of semiconductor manufacturing systems, as they are tailored to particular applications. As a result, the soft-sensing model development within the semiconductor industry has been constrained by its inflexibility and dependence on extensive prior knowledge of manufacturing mechanisms. This research endeavors to address these challenges by introducing a fully data-driven soft-sensing model for regression applications, focusing on wafer metrology systems. The objective is to offer a viable and practical solution that can be applied within the semiconductor sector.

In the domain of wafer metrology, soft-sensing regression models pertain to numerical models (statistical/machine-learning-based) that utilize real-time data from accessible sensors, like pressure and voltage data, to forecast quality indicators of importance. These soft-sensing models present an effective and economical approach in scenarios in which these indicators cannot be measured automatically or are only assessable through time-consuming, expensive, or sporadic follow-up metrology processes. Through the utilization of accessible sensor data, these models enable precise forecasting of the targeted quality indicators, leading to enhanced efficiency and effectiveness in the metrology processes. Soft-sensing models can be classified into two separate categories: *white-box* physical models and *black-box* data-driven models. Physical modeling relies on prior knowledge and typically concentrates on the ideal steady-state processing, rendering it unsuitable for handling intricate systems. In contrast, data-driven models leverage historical equipment and process data, allowing for calibration to achieve precise estimations of specific metrology variables. This capability enables pure data-driven virtual inspections of wafers, facilitating quality evaluations without the necessity of a physical examination.

Creating precise soft-sensing regression models is a multifaceted undertaking that demands thorough attention to numerous hurdles. Foremost among these challenges is elucidating the intricate connection between sensor readings and measurements, encompassing diverse time intervals. Furthermore, the level of precision needed for different types of wafers may differ, amplifying the intricacy of the task. Finally, the presence of incomplete data and measurement inaccuracies in the dataset further compounds the difficulty of constructing dependable models. Seagate factories, for example, collect extensive sensor data during wafer manufacturing to develop data-driven soft-sensing models. However, as the semiconductor manufacturing process becomes more complex, measuring key quality indicators using traditional metrology tools is becoming increasingly challenging and expensive. Developing a soft-sensing system to detect defective wafers can save both time and capacity for metrology tools.

The distinctive attributes of the wafer manufacturing data pose challenges when employing conventional physical models for regression. Thus, an alternative approach involving a nonlinear data-driven model becomes imperative. Data-driven models, such as Long Short-term Memory (LSTM) [1] network, GRU [2], and Transformer [3], are popular tools for tackling sequential data. LSTM-based models [1], widely applied in Natural Language Processing (NLP) tasks (e.g., text classification [4], machine translation [5] and speech recognition [6].), can handle sequential data of different lengths and overcome the vanishing gradient problem. Given the sequential nature of sensor recordings, it is natural to apply these NLP tools to soft-sensing regression task, where each *sensor value* in a time series corresponds to a *word* in a sentence in NLP.

In soft-sensing, there are applications of LSTM-related models in the penicillin fermentation process [7], the debutanizer column process [7,8], grinding classification process [9] and the wastewater treatment process [8], which demonstrate the capacities of the LSTM architecture in various industries. Although these LSTM-based soft-sensing regression models have been proposed for certain fabrication processes, their performance of general metrology in a real-world wafer manufacturing setting has not yet been investigated. Unlike other industries, wafer manufacturing has various measurement types, processes and precision requirements, and therefore requires the model to flexibly accommodate different circumstances. As such, this study proposes an LSTM-based soft-sensing regression model to forecast wafer metrology, with the aim of testing its performance in a real manufacturing process. This work seeks to answer the following research questions:

1. What are the challenges associated with real-world wafer manufacturing data, and which pre-processing techniques can be employed to convert raw and noisy wafer manufacturing sensor data into predictive insights?
2. How can the LSTM-based model be applied to predict the metrology values of wafers?
3. What objective functions can accommodate the varying precision requirements of the wafers?
4. How can the virtual metrology performance be evaluated when requirements differ for different wafer processing?

To address these questions, this study presents a thorough examination of the wafer soft sensing regression dataset while introducing a well-defined approach to its preprocessing. In addition, two distinct loss functions have been developed to accommodate the varying precision requirements of the wafers. Finally, the subjective evaluation criteria used by engineers have been translated into objective mathematical evaluation criteria to enhance the objectivity and rigor of the analysis.

The remainder of this paper is organized as follows. Section 2 summarizes some existing works related to our soft sensing regression problem. Section 3 introduces our model architecture and loss functions. Our data and our data preprocessing method are explained in Section 4. The experiments are described in Section 5. Finally, we present our discussions and conclusion in Section 6.

## 2. Related Work

A survey [10] conducted in the early stage on explored various regression approaches applied to VM, encompassing Simple Linear Regression, Partial Least Square Regression, Ridge Linear Regression, and Support Vector Regression [11]. Nevertheless, recent research in the domain of soft-sensing modeling has primarily concentrated on deep learning. In a recent survey paper [12], four key techniques in the field of soft sensing were highlighted: Autoencoder (AE) [13,14], Restricted Boltzmann Machine [15,16], Convolutional Neural Network (CNN) [17], and Recurrent Neural Network (RNN) [18].

Of these techniques, RNN-based or LSTM-based soft sensing models have found a lot of success with data that have strong sequential characteristics. LSTM-FCN [19] is a hybrid model that combines fully convolutional neural networks (FCN) features with LSTM features and uses dimension shuffle and dropout [20] to enhance the classification performance. To guide the learning process, SLSTM [7] incorporates prediction quality variables into the input, forget, and output gates of LSTM cells. On the other hand, DLSTM [9] utilizes the differences between the previous and current time steps as part of its inputs to the LSTM cells. Lastly, SBiLSTM [8] is similar to SLSTM [7] but uses a length-k window of quality information and is bidirectional.

In the wafer manufacturing setting, advancements in state-of-the-art soft-sensing models have recently been made, but with a focus on classification, which is distinct from our soft-sensing regression problem. One such model, Soft-sensing Transformer (SST) [21], utilizes a Transformer encoder [3] to demonstrate the similarities between sensor readings and text data. Another model, ConFormer [22], leverages multi-head convolution modules to achieve fast and lightweight operations while still being able to learn robust representations through multi-head design, similar to transformers. Soft-sensing Model Visualization [23] fine-tunes the model by adjusting the weights of input features based on misclassified examples. Finally, GraSSNet [24], a flexible Graph Neural Network [25,26] model, is suitable for semi-supervised settings. Soft sensing regression and classification are two distinct tasks with notable differences, particularly in the treatment of class imbalance. Soft sensing classification models may encounter issues with class imbalance, resulting in a biased model that performs poorly on minority classes. Conversely, soft sensing regression deals with a continuous target variable and does not require balancing classes, minimizing concerns regarding class imbalance. Our specific task involves training multiple types of wafers with varying precision requirements concurrently, which sets it apart from typical soft-sensing models. This unique characteristic necessitates the use of specialized loss functions.

## 3. Method

Our model uses sensor and categorical features as inputs, with the latter being textual variables derived from indicators of different measurement types and processes. The wafer may undergo multiple measurements at multiple points, and we use the median of those measurements, referred to as *meas_med*, as the target variable for our model’s prediction.

To handle sequential characteristics in data, we have opted to use LSTM to build our model, as explained in Section 3.1. In Section 3.2, we introduce two loss functions for our soft sensing regression task. We have observed that the typical $L_{2}$ loss function is unsuitable for our task, as diverse wafers often entail varying precision requirements.

### 3.1. Model Architecture

**Figure 1:**
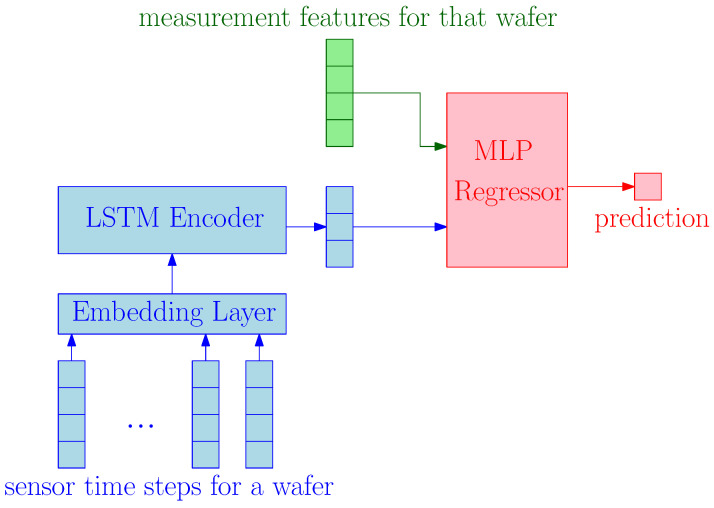
Long Short-term Memory (LSTM) model.

Figure 1 illustrates our approach to processing sensor data from a single wafer. To encode the sensor time steps into a fixed-dimensional vector, we use a one-layer LSTM encoder with an embedding layer. The encoder’s last cell state represents the encoded sensor vector. The embedding layer converts categorical and numerical variables into fixed-size vectors before feeding them into the LSTM structure.

To predict the target for a given wafer, we concatenate the features pertinent to the metrology tools, i.e., measurement features, with the encoded sensor vector and employ a Multi-Layer Perceptron (MLP). This approach reflects the chronological order of the wafer manufacturing process, in which sensors capture data before metrology tools measure the wafer.

### 3.2. Loss Functions

Our approach to soft-sensing modeling differs from traditional models, which aim to accurately predict a single target variable across all wafers. In contrast, our model considers the varying precision requirements of different types of wafers and aims to optimize predictions for each wafer type individually. To achieve this, we propose designing customized loss functions that account for the distinct precision levels required for each wafer type. This is a significant departure from traditional models that rely on a single loss function to optimize performance across all wafers. By tailoring our loss function design to the specific precision requirements of each wafer type, we can improve the overall accuracy of our predictions. Our paper focuses on jointly training these wafers while adapting to their unique precision requirements.

#### 3.2.1. Relative Error Loss

For ground truth *y* and prediction $\hat{y}$, the *Relative Error* is defined as

(1) $$\eta =\frac{|\hat{y}-y|}{\left|y\right|}$$

The Relative Error is a widely used metric for evaluating regression models. It measures the difference between the predicted value $\hat{y}$ and the ground truth value *y* as a fraction of |y|. However, we observed that the difference between upper and lower control limits is often small for ground truths with small absolute values, indicating the need for higher precision in such cases. To address this issue, we propose a modified loss function, denoted as $L_{RE}\left(\hat{y},y\right)$, defined as follows:

(2) $$L_{RE}\left(\hat{y},y\right)=\frac{|\hat{y}-y|}{\max(|y|,c)}$$

Here, *c* is a positive constant (set to 10 in our experiments) that allows us to avoid division by zero and prevent extreme values from dominating the loss. This modified loss function is a weighted $L_{1}$ loss, wherein larger weights are allocated to samples with smaller |y|. This weighting mechanism addresses the shortcomings of traditional $L_{1}$ or $L_{2}$ loss functions, making our proposed Relative Error Loss more effective at minimizing errors for small ground truth values.

#### 3.2.2. Normalized $L_{1}$ Loss

To generalize the Relative Error Loss, we can normalize each *meas_med* using two constants $b_{1}$ and $b_{2}$, where $b_{1}<b_{2}$, then train with $L_{1}$ loss. Ideally, $b_{1}$ and $b_{2}$ should be determined by some categorical variables within the measurement features. The formula used to normalize the target *y* is

(3) $$\tilde{y}=\frac{y-b_{1}}{b_{2}-b_{1}}$$

Given a model output $\hat{\tilde{y}}$, our Normalized $L_{1}$ loss function is

(4) $$L_{NL_{1}}\left(\hat{\tilde{y}},y\right)=\left|\hat{\tilde{y}}-\tilde{y}\right|$$

During prediction, we can transform our model output back to the original scale by using

(5) $$\hat{y}=\hat{\tilde{y}}*\left(b_{2}-b_{1}\right)+b_{1}$$

Intuitively, $b_{1}$ and $b_{2}$ should be similar to the lower and upper control limits mentioned in Section 4.2, but in reality people may change the lower and upper control limits from time to time. Therefore, the choice of $b_{1}$ and $b_{2}$ for each wafer is a crucial step. Compared to the Relative Error Loss, Normalized $L_{1}$ Loss is more general but requires the choice of $\left(b_{1},b_{2}\right)$ for each category.

## 4. Data

This section provides an overview of our datasets sourced from Seagate factories, as well as our data preprocessing techniques. To account for potential process drift in equipment status, we have included date and time information as input features for our model.

### 4.1. Sensor Data and Measurements Data

**Figure 2:**
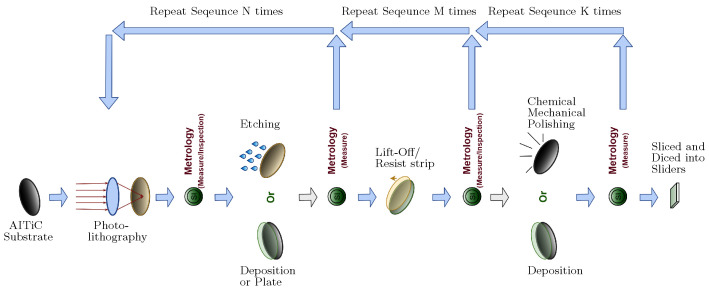
Workflow of wafer manufacturing. Figure accessed on 30 August 2021, available at https://github.com/Seagate/softsensing_data.

**Figure 3:**
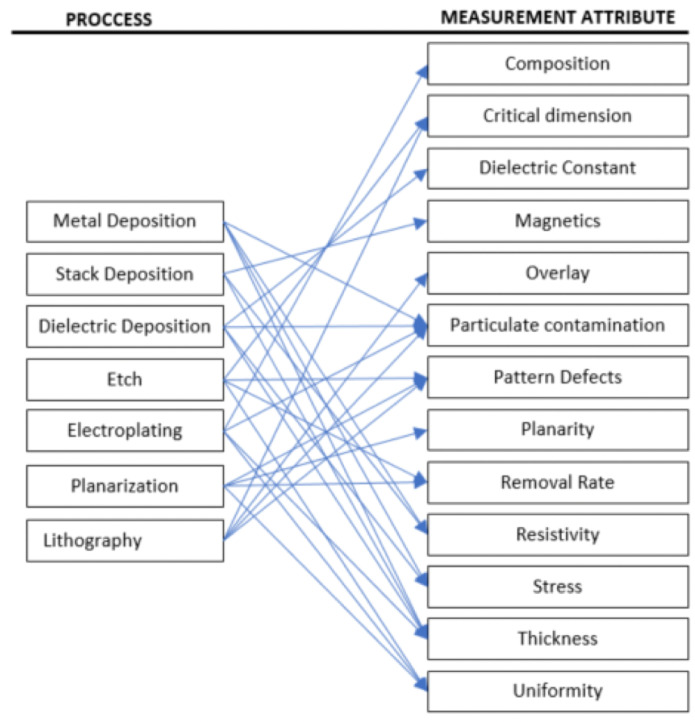
Processes and measurement attributes. Figure accessed on 30 August 2021, available at https://github.com/Seagate/softsensing_data.

In the manufacturing process as shown in Figure 2, a wafer undergoes various processing stages, such as lithography, etching, deposition, and polishing. At the completion of each processing stage, the wafer is subject to quality control inspection, which involves utilizing metrology tools to obtain several critical measurements that serve as key quality indicators (KQIs). Common KQIs obtained at different process stages are summarized in Figure 3, with the arrows pointing at the KQIs. Many KQIs are measured by more than one process stage, making it necessary to train the model jointly across different process stages and KQIs in order to utilize more information. This inspection process generates two mapping datasets, namely a sensor dataset and a metrology dataset. These datasets are essential for evaluating the wafer’s quality and ensuring that it meets the required standards. After removing duplicate samples, the sensor dataset now encompasses 1,301,234 rows and 144 columns, while the metrology dataset comprises 4,579,232 rows and 32 columns. It should be noted that both datasets include ID columns, specifically processing ID and product ID.

Each wafer can be uniquely identified by a unique pair of processing and product IDs. The sensor dataset, in addition to the *hard* sensor types of data, also contains the *soft contextual* data, including textual reports, such as textual information of the multi-stage manufacturing process, tools, processing modules, etc. These are referred to as categorical variables. There are seven textual categorical columns in the sensor dataset and eight categorical columns in the metrology dataset. Multiple metrology records measured at different inspection stages using different measuring methods are also obtained from each wafer.

### 4.2. Lower and Upper Control Limits

There are two datasets that are related to lower and upper control limits. One dataset provides two numbers *lcl* and *ucl* for each type of wafer. The other dataset contains the measurements data, which provides two numbers *targ_min* and *targ_max* in some rows. Both (lcl, ucl) and (targ_min, targ_max) are meant to be the lower and upper control limits, but *targ_min* and *targ_max* are usually more accurate than *lcl* and *ucl*. We set the lower and upper control limits using the following rules: if *targ_min* and *targ_max* exist, we assume them to be the lower and upper control limits; otherwise, we use *lcl* and *ucl* as the lower and upper control limits.

### 4.3. Pass/Fail Labels

The *passfail* column in measurements data contains different types of labels. Since we only predict *meas_med*, we only focus on predicting three types of labels: PASS, FAIL_AVG_HI and FAIL_AVG_LOW. FAIL_AVG_HI means the wafer failed because *meas_med* is higher than the upper control limit, and FAIL_AVG_LOW means the wafer failed because *meas_med* is lower than the lower control limit. Human inspection results are also provided for wafers with labels FAIL_AVG_HI and FAIL_AVG_LOW.

### 4.4. Data Preprocessing

**Figure 4:**
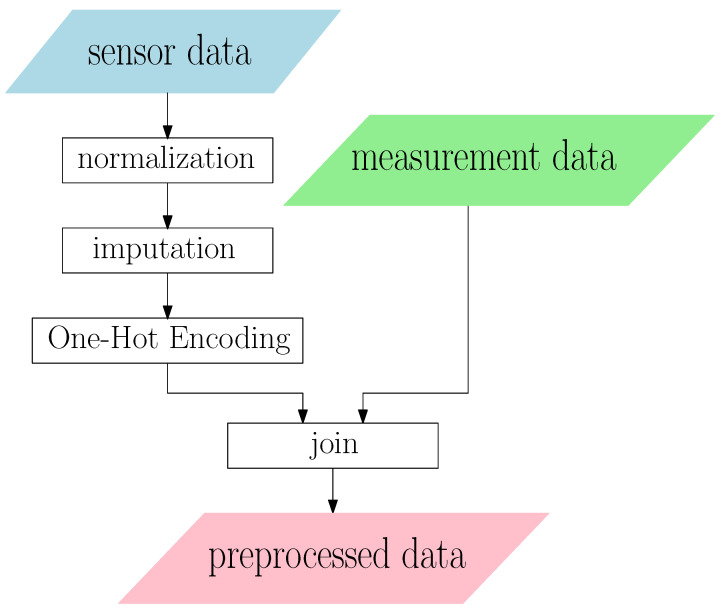
Data preprocessing flowchart.

**Figure 5:**
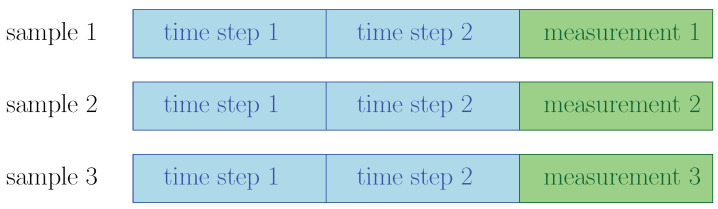
Join sensor data and measurement data.

Our data preprocessing is outlined in Figure 4. The data are split into training, validation and test sets by a chronological ratio of 7:2:1. Due to the constraint of storage and computing resources, we only focus on the 33 most common *(KQI, TYPE)* of wafers. Because different columns have different scales, we use linear transformations to normalize each feature in the sensor training set to the interval [0,1], then use the same linear transformations to transform the validation and test set. The missing values in the sensor readings are imputed with the medians. Those samples with *meas_med* outside the range [−1, 1000] are dropped in the training set because they are likely to be measurement errors. The dropped samples consist of less than 1% of the whole dataset. One-Hot Encoding are performed on the five categorical columns in the sensor data and five categorical columns in the measurements data. After that, we have 267 and 552 features in sensor and measurement data, respectively. The sensor data and measurements data are joined before model training. An example can be found in Figure 5, where in this example, the wafer has two time steps and three measurements. The *join* operation concatenates these two time steps in a row and joins them with every one of the three measurements, resulting in three samples after preprocessing.

## 5. Results

### 5.1. Evaluation Metrics

#### 5.1.1. Error Grouping

Our target *y*, i.e., *meas_med*, is a continuous distribution on [−1, 1000], and sometimes *y* is close to zero. When |y| is small, *Relative Error*η can easily become very large and can be meaningless. For those measurement values close to zero, the processing engineers validate that the *Absolute Error* $\epsilon =|\hat{y}-y|$ is of much importance when it comes to processing monitoring and adjustments. So, we propose the following grouping criteria for prediction errors:

- Group 1: η<1% or ϵ<0.1.
- Group 2: η<5% or ϵ<0.5, and not in Group 1.
- Group 3: η<10% or ϵ<1, and not in Group 1 or 2.
- Group 4: η<50% or ϵ<5, and not in Groups 1–3.
- Group 5: η<100% or ϵ<10, and not in Groups 1–4.
- Group 6: not in Groups 1–5.

Apparently, a group with smaller index is a better group. We report the grouping results in our experiments, and define ‘decent predictions’ as those predictions in group 1 and group 2.

#### 5.1.2. Recall and False Positive Rate

The *passfail* column and human inspection results give us insights into the pass or fail status of wafers, as introduced in Section 4.3. In our evaluation, we define a ‘fail wafer’ as a wafer which satisfies all the following conditions:

- The value in its *passfail* column is FAIL_AVG_HI or FAIL_AVG_LOW.
- The result of human inspection is also a fail.
- Its *meas_med* is either larger than the upper control limit or smaller than the lower control limit.

The number of fail labels in our dataset is much smaller than the number of pass wafers. Fail wafers are regarded as positive samples. The recall rate of fail wafers and the false positive rate are our evaluation metrics.

### 5.2. Compare Two Losses

In this subsection, we compare the Relative Error Loss with the Normalized L1 Loss. We group the data by *(KQI, TYPE, stage)* and call each group a normalization group. In the Normalized L1 Loss, we normalize *meas_med* within each *(KQI, TYPE, stage)* normalization group, then train with L1 loss as we mentioned in Section 3.2.2. There are two other categorical variables of lesser importance: *equipid* and *prod*. We do not include *equipid* and *prod* in the normalization to prevent an excessive proliferation of normalization groups. Using *(KQI, TYPE, stage)*, we establish 74 normalization groups in the entire training set, a quantity we deem appropriate compared to our sample size. Within each *(KQI, TYPE, stage)* normalization group, the pair $\left(b_{1},b_{2}\right)$ introduced in Section 3.2.2 is chosen based on having the smallest difference between the upper and lower control limits.

#### 5.2.1. LSTM-Small Model

Our model architecture is explained in Section 3.1. When we train on only one *(KQI, TYPE)*, we use relatively fewer parameters to define a LSTM-small model. Later, when we train on all data, we define a LSTM-large model with more parameters. In the LSTM-small model, our input size and hidden size of LSTM are both 128, where the input size of LSTM is the output size of the embedding layer. In the MLP regressor, we have two hidden layers, each with 256 hidden units. In total, there are approximately 0.4 million parameters in the LSTM-small model. Our activation function in the MLP regressor is ReLU. We stop the training if we observe no improvement in the validation loss for more than 10 continuous epochs. Our optimizer is Adam with a learning rate of 0.0001. After some hyperparameter tuning, we set the batch size to be 16. We choose the model with the best validation loss.

Table 1 summarizes the test set results when we train our LSTM-small model on *KQI* KQI-1 and *TYPE* TYPE-1. We choose this *(KQI, TYPE)* because it is one of the most common *(KQI, TYPE)*s. In the table, *RE* means the training loss is the Relative Error Loss. And *NL1* means the Normalized $L_{1}$ Loss. Each row in the last column contains a list of six numbers, representing the number of test samples in each group under our grouping criteria. According to our experiments, Relative Error Loss gives us better results. Since we have already defined predictions within group 1 and group 2 as decent predictions, we have 5413/5749≈94.16% decent predictions using RE loss, while we only have 5270/5749≈91.67% decent predictions using NL1 loss.

#### 5.2.2. LSTM-Large Model

We train all 33 different *(KQI, TYPE)*s jointly to see if we can improve the results. A much larger model called the LSTM-large model is used. Its input size and hidden size of LSTM are both 1024. Its MLP regressor still has two hidden layers but each hidden layer has 2048 units. The LSTM-large model has approximately 16 million parameters in total. We still use batch size 16 and the Adam optimizer with a learning rate of 0.0001.

The results are displayed in Table 2. Based on Relative Error Loss, 23074/28134≈82.01% predictions are decent predictions. This is worse than the 94.16% prediction for the LSTM-small model on (KQI-1, TYPE-1), perhaps because (KQI-1, TYPE-1) is a relatively easy *(KQI, TYPE)*. Based on the Normalized L1 Loss, we have 22418/28134≈79.68% decent predictions, which is still worse than the result based on Relative Error Loss. However, if we only care about the number of predictions within group 1, the model trained with Normalized L1 Loss has 12812 samples in group 1, which is better than the 10763 samples for the model trained with Relative Error Loss. Another experiment we find interesting is one that uses the same LSTM-large model trained on all 33 *(KQI, TYPE)*s but tests only on (KQI-1, TYPE-1). We have 5419/5749≈94.26% decent predictions achieved via *RE* Loss and 5417/5749≈94.23% decent predictions achieved via *NL1* Loss; see Table 3. Both 94.26% and 94.23% are better than their counterparts in the LSTM-small model; this is because we have much more training data when we train all 33 *(KQI, TYPE)*s jointly.

Upon comparing the percentage of decent predictions, we find that the Relative Error Loss slightly outperforms the Normalized L1 Loss within our settings, but the Normalized L1 Loss possesses its own advantages. Following the same normalization procedure, it is straightforward to replace the L1 loss with L2 loss or Huber loss. Therefore, the Normalized L1 Loss is more general. Additionally, the Normalized L1 Loss incorporates the lower and upper control limits into the model, while the Relative Error Loss does not. This characteristic of the Normalized L1 Loss is beneficial when precise lower and upper control limits are available.

### 5.3. Pass/Fail Evaluation

The definition of a fail wafer can be found in Section 5.1.2. Under that definition, we only have 162 fail wafers among 550,239 training samples. Using the LSTM-large model trained on all *(KQI, TYPE)*, and predicting a wafer to be a fail wafer if and only if the predicted meas_med $\hat{y}$ is outside the interval $\left(b_{1}^{*},b_{2}^{*}\right)$, we achieve a recall rate of 0.3580 and a false positive rate of 0.01434. The $b_{1}^{*}$ and $b_{2}^{*}$ are the lower and upper control limits introduced in Section 4.2.

To sacrifice the false positive rate for a better recall rate, we apply the following trick here: considering a constant 0<f<0.5, and $r=b_{2}^{*}-b_{1}^{*}$, we predict the wafer as a fail wafer if and only if

(6) $$\hat{y}\notin \left(b_{1}^{*}+f*r,b_{2}^{*}-f*r\right)$$

When *f* increases, both the recall rate and false-positive rate of the model are expected to increase, given that more wafers are predicted to be fail wafers. The results are presented in Table 4, revealing that a f=0.35 yields a recall rate above 0.8. The false-positive rate at this point is 0.2981. If an 80% recall rate suffices, Table 4 illustrates that we now only need to apply metrology tools on approximately 30% of the wafers where $\hat{y}\notin \left(b_{1}^{*}+0.35r,b_{2}^{*}-0.35r\right)$. Due to the presence of potentially misclassified fail wafers in our dataset, the actual recall rate should be higher. With the accumulation of more test data in future endeavors, our results will become more accurate.

## 6. Discussion and Conclusions

In this paper, we address the challenges of handling real-world manufacturing data by presenting a preprocessing method that considers both numeric sensor recordings and textual recordings concurrently. To cater to the unique characteristics of wafer manufacturing data, we propose an LSTM-based model that predicts the metrology values of wafers. Our model utilizes two distinct loss functions, the Relative Error Loss and the Normalized L1 Loss, which accommodate the varying precision requirements of the wafers. We evaluate the performance of our model using specific criteria for soft sensing regression. Our results demonstrate that deep learning techniques can effectively forecast metrology measurements, thereby reducing the reliance of factories on metrology tools and resulting in significant time and energy savings. Overall, our study highlights the potential benefits of leveraging deep learning in manufacturing settings.

To improve the model’s effectiveness in future studies, we should prioritize the removal of outliers from the dataset. Our analysis identified erroneous measurements that could compromise both the accuracy of the model’s training and evaluation. In addition, we recommend considering transfer learning techniques by leveraging soft sensing data from various metrology tool sets within Seagate. By doing so, we can enhance the model’s capacity to make more robust predictions and improve its generalization capabilities.

## Figures and Tables

**Table 1:** LSTM-small model on (KQI-1, TYPE-1).

Model	Loss	Relative Error Grouping
LSTM-small	RE	[2554, 2859, 257, 68, 10, 1]
LSTM-small	NL1	[2544, 2726, 319, 100, 36, 24]

**Table 2:** LSTM-large model on all data.

Model	Loss	Relative Error Grouping
LSTM-large	RE	[10,763, 12,311, 2122, 2899, 32, 7]
LSTM-large	NL1	[12,812, 9606, 2071, 3458, 98, 89]

**Table 3:** LSTM-large model on (KQI-1, TYPE-1).

Model	Loss	Relative Error Grouping
LSTM-large	RE	[2380, 3039, 231, 78, 19, 2]
LSTM-large	NL1	[2626, 2791, 210, 55, 42, 25]

**Table 4:** Recall and false-positive rate.

*f*	Recall	False Positive Rate
0.0	0.3580	0.01434
0.1	0.4506	0.07219
0.2	0.4938	0.1084
0.3	0.6975	0.2075
0.35	0.8086	0.2981
0.4	0.8395	0.4321

## Data Availability

Data sharing is not applicable to this article.
